# Benefits of Repeated SARS-CoV-2 Vaccination and Virus-induced Cross-neutralization Potential in Immunocompromised Transplant Patients and Healthy Individuals

**DOI:** 10.1093/ofid/ofae527

**Published:** 2024-09-09

**Authors:** David Hauser, Lorena Urda, Christopher Lang, Christian Mittelholzer, Fabian Otte, Enja Kipfer, Yuepeng Zhang, Martin Lett, Christiane Schebitz, Roman-Ulrich Müller, Wilfried Klimkait, Thomas Klimkait

**Affiliations:** Molecular Virology, Department of Biomedicine, University of Basel, Basel, Switzerland; Molecular Virology, Department of Biomedicine, University of Basel, Basel, Switzerland; Molecular Virology, Department of Biomedicine, University of Basel, Basel, Switzerland; Molecular Virology, Department of Biomedicine, University of Basel, Basel, Switzerland; Molecular Virology, Department of Biomedicine, University of Basel, Basel, Switzerland; Molecular Virology, Department of Biomedicine, University of Basel, Basel, Switzerland; Molecular Virology, Department of Biomedicine, University of Basel, Basel, Switzerland; Molecular Virology, Department of Biomedicine, University of Basel, Basel, Switzerland; Gemeinschaftspraxis Martinstrasse, Kinder- und Jugendmedizin, Olpe, Germany; Department II of Internal Medicine and Center for Molecular Medicine Cologne, University of Cologne, Cologne, Germany; KfH-Nierenzentrum, Heilig-Geist-Gesundheitszentrum, Köln-Longerich, Germany; Molecular Virology, Department of Biomedicine, University of Basel, Basel, Switzerland

**Keywords:** immunodeficiency, kidney transplant, live neutralization, SARS-CoV-2, transplant patient, vaccination

## Abstract

**Background:**

Current COVID-19 vaccines primarily target the Spike protein of defined virus variants, offering limited protection against emerging variants in immunocompetent individuals. Similarly, protective immunity following natural SARS-CoV-2 infection is variable and of short duration, raising concerns about immunocompromised individuals' vaccination strategies.

**Methods:**

This prospective multicenter study examined 66 sera from 59 immunocompromised and 451 sera from 215 immunocompetent individuals from different pandemic periods. We establish and validate a live virus-based neutralization assay to determine the virus-inactivating potential against ancestral and current SARS-CoV-2 isolates.

**Results:**

Our virus-based neutralization assay demonstrated superior performance over surrogate neutralization assays. We found strong but transient immunity after complete vaccination schemes, with single doses providing minimum neutralization, regardless of vaccine type. Combining vaccination-induced immunity with SARS-CoV-2 infection before or after vaccination yielded higher neutralizing titers than vaccination or infection alone, consistent across both study groups. Additional doses after a full vaccination course restored neutralization levels.

**Conclusions:**

Potentially protective SARS-CoV-2 neutralization is reliably induced in immunocompromised individuals by prior attenuation of immunosuppression. First-generation vaccines protect against various SARS-CoV-2 variants in immunocompetent individuals, with effective cross-neutralization demonstrated up to the Delta variant but largely absent for later Omicron variants. Continuous vaccine updates are necessary to address emerging SARS-CoV-2 variants.

## Teaser

This study evaluated the impact of repeated vaccination and SARS-CoV-2 infection on the virus neutralization capacity in immunocompromised and immunocompetent individuals during different episodes of the pandemic. Neutralization over time was categorized by age, immunization history, and vaccination status.

After the SARS-CoV-2 outbreak in late 2019 quickly reached pandemic dimensions, the World Health Organization declared an end to the global emergency status for COVID-19 on 5 May 2023 [1]. This timeframe aligns with the historical duration of other viral outbreaks, which range from 1 to >3 years for epidemic pathogens [2]. The shift to an endemic phase often coincides with the appearance and accumulation of mutations in the pathogen's genome during periods of growing herd immunity in affected populations. This leads to declining immune protection [3] and a rise in (re-)infection cases with mutated virus variants capable of evading existing immunity [4]. Consequently, SARS-CoV-2 continues to pose a global threat, with mutated variants likely triggering new or sustained waves of infections and the reemergence of COVID-19 [5, 6].

Assessing protection levels and vaccine efficacy requires determining whether vaccines based on a previously circulating variant can control newly emerging virus variants. The initial global spread of SARS-CoV-2 was driven by a homogeneous ancestral SARS-CoV-2 strain (“Wuhan”) [7]. The stability of this variant, dominant for many months, was attributed to SARS-CoV-2's proofreading ability [8] and a relatively low mutation rate [9]. Later variants of concern (Alpha, Beta, Delta, and Omicron) with specific genomic mutations triggered new outbreaks globally [7]. Omicron, emerging in India in early 2021 and South Africa in November 2021, gave rise to regional subvariants [10], including Omicron XBB.1.5., even during large international vaccination campaigns. The Omicron variants exhibit multiple unique mutations, particularly in the viral Spike (S) gene [11, 12], which correlate with the virus's increased ability to evade immune protection elicited by vaccination or prior SARS-CoV-2 infection [13, 14]. Fortunately, the recent Omicron variants possess relatively low pathogenicity compared to earlier waves. Whether these virus variants have lower pathogenic potential or if population-wide immunization has led to a generally milder infection course remains unclear [15, 16]. Although hybrid immunity (ie, combinatorial immunity elicited by both an infection and vaccination event) can be beneficial, the remaining risks of infection and severe COVID-19 largely depend on the personal immune constitution and the variant of the infecting SARS-CoV-2 [17]. Mathematical models predicting hybrid immunity suggested independence of vaccination and infection with no synergy [18]. Thus, a possible direct link continues to be disputed.

Lower vaccine response and concern of viral escape are especially concerning in the older population and individuals with compromised immune function. Because protective immunity is critical for these vulnerable populations, assessing vaccination responsiveness and the possible impact of hybrid immunity is of great medical interest. For the elderly, earlier reports remain controversial, either stating that, as expected, immunity and T-cell response inversely correlate with age [19] or showing that age effects were most prominent after the first vaccination but less or not critical after follow-up vaccinations [20].

Immunocompromised individuals are often underrepresented or excluded from large vaccine trials [21, 22]. Studies assessing antibody response to different vaccines in immunocompromised individuals (eg, transplant patients or persons with HIV) lack predictive information on response quality [23, 24]. Further research is needed on the durability and broadness of vaccine protection against new viral variants of clinical importance.

During the COVID pandemic, no binding guidelines for optimal vaccination strategies for transplant patients or those under immunosuppressive therapy were available. Pretreatment protocols to enhance vaccine responsiveness were also missing. Treating physicians were under extreme pressure to protect their patients from SARS-CoV-2 and save the lives of a very vulnerable group who were unable to mount a normal immune response. Furthermore, this was at a time when the only available treatment with antibodies was increasingly losing efficacy, no promising new treatment options for severe COVID were in sight, and some of the newer therapies were approved solely as prophylactics. Especially in this group of affected individuals, immunosuppression generally leads to a poor vaccine response [25], and suitable enhancement strategies are strongly advocated. Treatment centers, including the participating medical center, applied the German standard protocols outlined in the “Kidney Disease: Improving Global Outcomes Clinical Practice Guidelines” [26] to reduce immunosuppressive pressure around vaccination (ie, some weeks before a vaccine shot). When no immune response was noted after repeated vaccinations, a change was introduced after the third or fourth vaccinations: the immunosuppressant was reduced to 50% or completely paused for patients with favorably stable transplant parameters. This strategy was expected to provide a basis for a better immune response to the SARS-CoV-2 vaccine.

To assess the effects of vaccination strategies, infection events, and demographics in vulnerable populations, this multicenter study compared serum samples from immunocompromised kidney transplant patients and immunocompetent individuals. Study subjects were immunized with mRNA, vector-based vaccines, or their combination, and/or reported SARS-CoV-2 infection episodes. Serum was collected between 2021 and 2023, and virus neutralization was determined using live virus stocks of different SARS-CoV-2 variants in a newly established live-neutralization assay.

## METHODS

### Study Approval

The human subjects in this study were enrolled under study-ID No. 2022–00303, approved in March 2022 by the Ethikkommission Nordwest- und Zentralschweiz, and in April 2022 by both the Ethikkommission der Ärztekammer Westfalen Lippe (No. 2022-165-b-S) and the Ethikkommission der Ärztekammer Nordrhein (No. 2022060). The sponsor and main investigator of the study is Thomas Klimkait, University of Basel, with Nina Khanna, University Hospital of Basel, acting as a second local investigator. With the final preapproval documents, the same as those submitted to the respective national authorities, we ensured that local sampling could start as early as possible during the most critical early phase of the pandemic in 2021. Volunteers invited by study centers received information about the process and preliminary consent; they were then asked to donate a serum sample, which was strictly and confidentially stored at the local site. Only after official approval and with the signed consent form were samples shipped to the central diagnostic site for analysis.

### Clinical Context of Specimens

This study recruited 2 cohorts: 1 of immunocompetent individuals (COMP) from 4 centers in Switzerland and Germany and another of immunocompromised (SUP) kidney transplant patients from Germany, who received immunosuppressants. Participants donated 1 single or longitudinally several serum samples, collected on an average of 3 to 4 months after vaccination or infection. Serum samples with a maximum of 5 vaccinations were obtained, with mRNA- or adenovirus-vector-based vaccines. Infection events, either real time-polymerase chain reaction–confirmed or self-reported, were recorded, in some cases within a few months after the last immunization (Table 1).

**Table 1.** Participants' Characteristics and Vaccination History

General Information	COMP	SUP
No. of samples (individuals)	451 (215)	66 (59)
Gender: female samples (individuals)	256 (123)	25 (23)
Gender: male samples (individuals)	195 (92)	41(36)
Age of individuals in years (mean)	2–87 (42)^a^	30–83 (59)
*Vaccine type*	…	…
AstraZeneca/ChAdOx1, no. of samples (%)	124 (12)	1 (0.5)
BioNTech/Comirnaty, no. of samples (%)	599 (58)	176 (87)
Janssen/Ad26.COV2.S9, no. of samples (%)	92 (9)	-
Moderna/Spikevax, no. of samples (%)	215 (21)	26 (13)
*Verified COVID*	…	…
COVID, no. of individuals (%)	104 (48)	27 (46)
Hospitalization, no. of individuals (%)	3 (1.4)	6 (10)
COVID in individuals with 3–4 vaccinations (%)	59 (27)	17 (29)
COVID in individuals with 3–4 vaccinations within 3 mo after last vaccination (%)	45 (21)	5 (8)
*Sampling timepoint after immunization*	…	…
Sampling after last immunization in months (mean)	0.1–17 (3.3)^b^	0.4–13.4 (3.8)^c^
Sampling after last vaccination in months (mean)	0.1–13.6 (3.2)	0.4–11.3 (3.7)
Sampling after last infection in months (mean)	0.3–17 (2.9)	0.9–13.4 (4.0)
*No. of samples tested against virus strain*	…	…
A, no. of samples (mean/median titer)	414 (1457/640)	66 (710/120)
D, no. of samples (mean/median titer)	434 (1429/640)	66 (1025/80)
BA.1, no of samples (mean/median titer)	391 (615/160)	66 (202/80)
XBB.1.5, no of samples (mean/median titer)	123 (47/40)	10 (124/80)

Abbreviations: COMP, immunocompetent individual; SUP, immunocompromised individual.

^a^Two samples were excluded from this category because of missing data.

^b^Three samples were excluded from this category because of missing data.

^c^One sample was excluded from this category because of missing data.

### Procedure for Immunocompromised Study Participants

All immunosuppressive procedures were according to the “Kidney Disease: Improving Global Outcomes clinical practice guideline for the care of kidney transplant recipients” issued by the European Society for Organ Transplantation [26]. Of note, the clinical situation of pre- and posttransplant patients is, beyond the need for a functional organ, largely characterized by individual comorbidities and a rather unique immunological situation that often requires attention and personalized medication. This leads to a clinical situation that can rarely be managed by generalizable standard-of-care protocols. Immunocompromised patients, especially those with no measurable SARS-CoV-2 antibody response after the first cycles of vaccination as recommended, were immunologically “preconditioned” by lowering immunosuppression before the next vaccine shot. A confirmed stable transplant function and stable creatinine profile contributed to defining a low immunological risk. Accordingly, 1 week before a fourth vaccination, the proliferation inhibitor mycophenolic acid (or its prodrug mycophenolate-mofetil) was reduced to 50% of the original dose or paused. Three weeks after the fourth vaccine shot, immunosuppressive agents were readjusted to the normal dose. This protocol reflects procedures suggested by others [27, 28].

### Sampling

One tube of venous blood was drawn into a native blood collection tube (Sarstedt). After coagulation, the tube was centrifuged, the serum aspirated and aliquoted in 1.5-mL tubes. For analysis within days, serum aliquots were stored at 4°C; long-term storage was at −20°C.

### Live Virus Neutralization Assay

For a detailed protocol, see Supplementary Materials. In brief, sera in 1:2 dilution steps and infectious virus were added to cells seeded in 96-well plates. 3–4 days later, plaque formation was analyzed after cell fixation and crystal-violet staining. For the enumeration of viral plaques, the automated CTL software was used. Neutralization titers are reported based on the dilution step achieving more than 50% viral inhibition. To follow the antiviral response to viruses prevalent during different periods of the pandemic, most sera were tested against ancestral, Delta, and Omicron BA.1.5 variants, with a subset tested against the more recent Omicron variant XBB.1.5.

### SARS-CoV-2 Strains

Virus stocks originate from clinical isolates. The ancestral strain (Clinical isolate Muc-1, a Wuhan-1-type virus isolate) was provided by G. Kochs, University of Freiburg, Germany. Virus variants Delta B1, Omicron BA.1, and Omicron XBB.1.5 are sequence-verified local clinical isolates from nasopharyngeal aspirates of human donors who had given their informed consent (approval by Ethikkommission Nordwest- und Zentralschweiz No. 2022-00303). For details on viral propagation and handling, see Supplementary Materials.

### Statistical Analysis

Appropriate statistical tests were chosen based on sample size and are indicated in individual figures.

## RESULTS

### Validation of the Test System

To assess virus inactivation, we established a live virus neutralization assay (VNA) using SARS-CoV-2, with titrated stocks of clinically relevant variants: ancestral (A), Delta (D), Omicron BA.1 (BA.1), and Omicron XBB.1.5 (XBB). This assay has the advantage of quantifying antibody titers against infectious virus. Commercial semiquantitative enzyme-linked immunosorbent assay (ELISA) tests measure “binding antibody titers” to epitopes in the receptor binding domain of S in competition with the ACE2 receptor as a surrogate for virus neutralization. Comparison between tests (GenScript, EuroImmun, and VNA) shows a clear correlation, but the live neutralization test provided a broader neutralization spectrum and superior test sensitivity for high-titer sera. Near the detection threshold of the surrogate tests, virus neutralization was still able to provide reliable information on a person's immunization status down to serum titers of 1:40 (Supplementary Figure 1*A* and *B*).

For immunocompromised patients, the correlation of neutralization titers (measured by DiaSorin and VNA) decreased with increasing genetic distance of SARS-CoV-2 variants. This highlights the benefits of a live virus neutralization assay against different variants (Supplementary Figure 1*C*).

Because virus neutralization can principally be facilitated by various SARS-CoV-2 proteins beyond the S protein [29, 30], differences in titer can stem from the activity against other SARS-CoV-2 proteins, as shown with an in-house ELISA against Nucleocapsid protein (N) with possibly neutralizing [31] or potentially exacerbating consequences [32, 33] and against the Membrane protein (M) [34]: 4 of the 6 participants had elevated responses against N after infection/vaccination (Supplementary Figure  *1D*).

### Titer Dynamics Over Time Based On Epidemiological and Immunological Parameters

**Figure 1.**
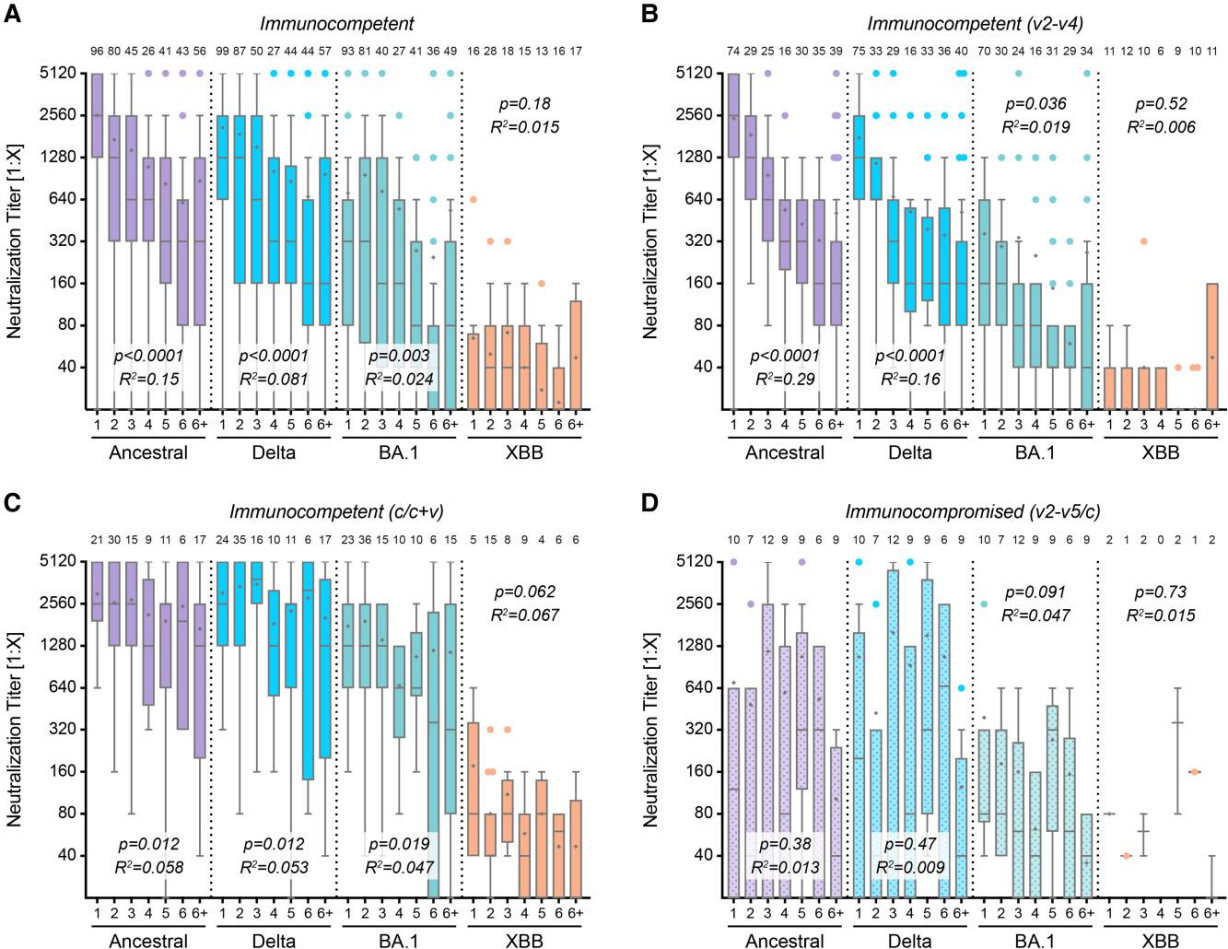
Neutralization titer over time. Titers against the ancestral Wuhan isolate, the Delta, Omicron BA.1, or Omicron XBB.1.5 variant, grouped in months (months 1–6 and >6 m [6+]) after vaccination (v) or infection (c). *A*, All immunocompetent (COMP) individuals who had been vaccinated or were convalescent (v1–4 and c/c + v). *B*, Subgroup of COMP individuals with 2–4 vaccinations (v2–4), or (*C*) subgroup of convalescent or mixed immunization status (c/c + v). *D*, Subgroup of immunocompromised (SUP) individuals with 2–5 vaccinations, convalescent or mixed immunization status (v2–5 and c/c + v). Data are presented as box plots showing the median, mean (+), 25th to 75th percentiles with whiskers, and outliers plotted based on Tukey, the corresponding number of samples is indicated above each box plot. Linear regression-based *P* values show deviation from slope = 0, the goodness of fit is indicated with *R*^2^.

We used the live virus neutralization assay to analyze titers against all variants based on the time of sampling after vaccination or infection. Immunocompetent individuals exhibit a decline over time for ancestral, Delta, and Omicron BA.1 strains, consistent with earlier reports [3, 35, 36] (Figure 1*A*). For Omicron XBB.1.5, our data confirm lower neutralization titers overall, and very low levels of cross-protection by the vaccines, all developed against earlier non-XBB variants (ie, the S of the ancestral alone or combined with S of the Omicron BA.1 virus) (Figure 1*A* and *B*). Convalescent individuals showed slower decay rates over time and higher titer levels, except for Omicron XBB.1.5, because of sampling occurring before virus circulation of Omicron XBB.1.5 (Figure 1*C*).

Comparing specimens from immunocompromised (Figure 1*D*) with the same category of immunocompetent (Figure 1*A*) individuals, titers as well as mean values were either much lower or negative for the immunocompromised group. This suggests that, because of lower immune competence, a higher number of booster vaccinations or convalescent episodes is needed to reach protective levels or, in some instances, even similar titers as immunocompetent individuals. In contrast to the overall titer, no significant decay over time was noted for the immunocompromised samples. This was somewhat unexpected because the lower level of immunological response appeared suggestive of a poorer protection level.

To ensure a full immunization response, only samples taken at least 2 weeks after vaccination or infection were included in the analysis. The upper limit of 4 months was defined according to higher variability in months 5, 6, and 6+ (Figure 1*A–C*). Additionally, we could not exclude a higher likelihood of inapparent infections with increasing sampling time after infection/vaccination. Based on this, further analysis includes only specimens sampled between 2 weeks and 4 months after immunization.

### Effects of Gender and Age On Neutralizing Capacity

In vaccination studies against viral diseases, differences in response between men and women have been observed. In this SARS-CoV-2 study, we found no significant gender differences in neutralization capacity against all measured SARS-CoV-2 variants (Supplementary Figure 2*A*).

**Figure 2.**
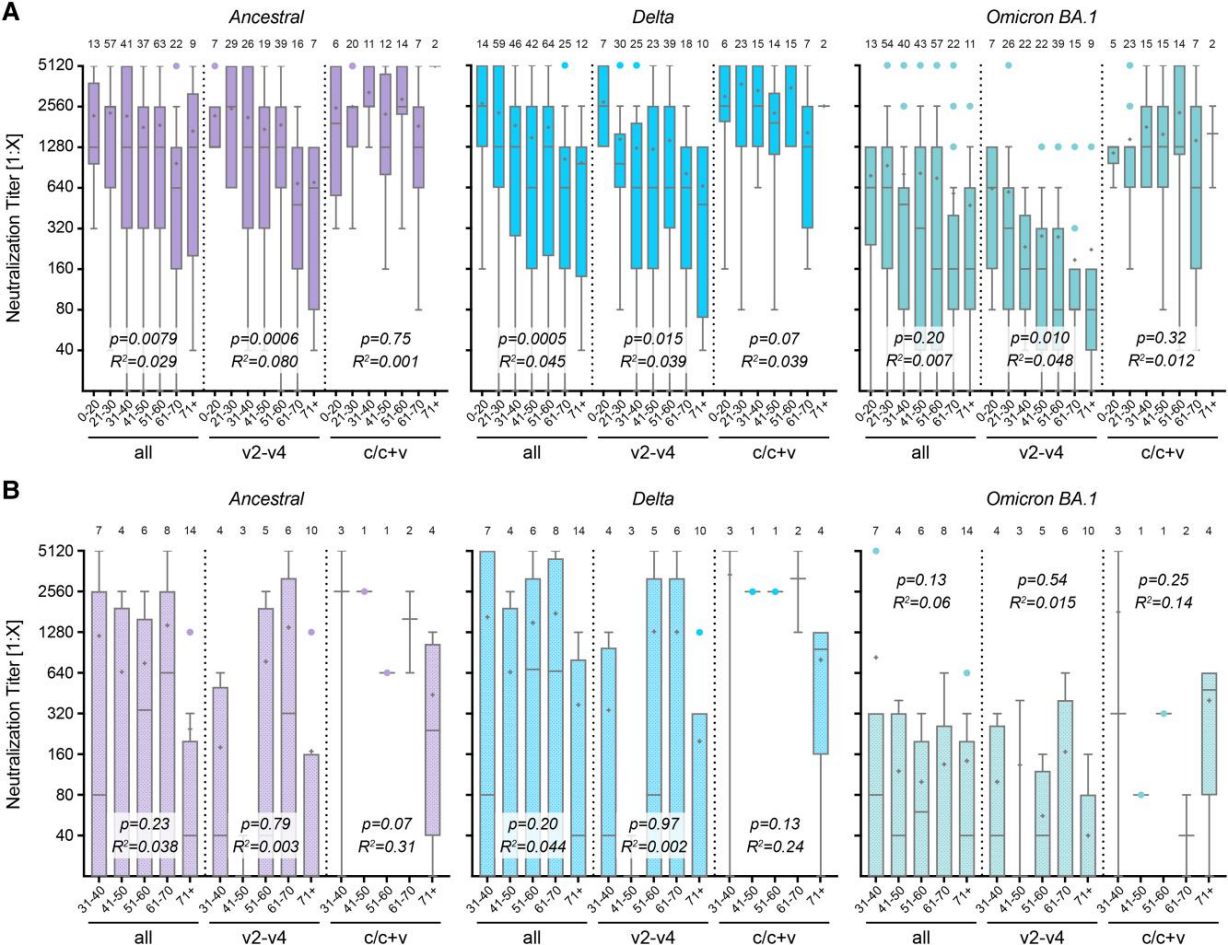
Age dependence of neutralization capacity. Neutralization titers against SARS-CoV-2 variants during periods from 0.5 to 4 months post-immunization. Samples were grouped in age intervals for COMP (*A*) and SUP (*B*) samples, either with all samples, including those after 1 single vaccination (first group, “all”), after 2–4 vaccinations (v2–v4), or after infection or infection and vaccination (c/c + v) as indicated. Titers are measured against the ancestral Wuhan virus isolate, the Delta, or Omicron BA.1 variant. Data are presented as box plots showing the median, mean (+), 25th to 75th percentiles with whiskers, and outliers plotted based on Tukey, the corresponding number of samples is indicated above each box plot. Linear regression-based *P* values show deviation from slope = 0, the goodness of fit is indicated with *R*^2^.

Earlier studies have reported a decline in antibody titers with increasing age [37, 38]. In our analysis, we grouped samples by age as follows: younger than 20 years, in 10-year intervals between 20 and 70 years, and older than 70 years. We confirm an age-related decrease in the immune response for all tested virus variants in samples from individuals with at least 1 immunization event (“all”, Figure 2*A*). This decrease was not observed in immunocompetent individuals with an infection event or a mixed immunization status, in contrast to vaccinated-only individuals. Neutralization titers in convalescent individuals were significantly higher when compared to vaccinated individuals in the age groups 21–30, 31–40, and 51–60 years against Delta (*P* < .0001, *P* = .0007, and *P* = .0002, respectively; 2-way analysis of variance (ANOVA) followed by Bonferroni's test), and in all age groups from 21 to 70 years against Omicron BA.1 (*P* = .022, *P* = .0001, *P* = .0016, *P* < .0001, and *P* = .048 resp., Figure 2*A*). Against Omicron XBB.1.5, there was no significant decrease for all samples and the vaccinated-only group; the reported age-related increase for convalescent individuals is based on a very small sample size (Supplementary Figure 2*B*).

As expected, the drug-based immune reduction or suppression in transplant patients led to lower antiviral titers than in age-matched immunocompetent participants. No age-related decrease was observed in immunocompromised individuals, regardless of the virus variant or immunization group (Figure 2*B*). Although this observation was not established in the literature, it should be noted that all immunocompromised participants had been vaccinated multiple times before enrollment to provoke measurable antibody titers. We cannot exclude that this intervention contributed to a less age-dependent overall response.

### Hybrid Immunity in Immunocompetent and Immunocompromised Individuals

**Figure 3.**
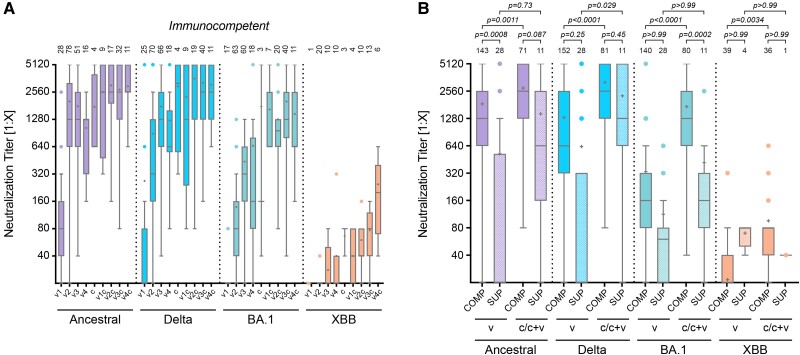
Immunity after vaccination, infection, or hybrid status. *A*, Neutralization titers of immunocompetent individuals (COMP) after vaccination (v) only (v1, v2, v3, and v4), infection only (c), or hybrid status, regardless of the sequence of events (v1c, v2c, v3c, and v4c). *B*, Comparison of neutralization titers of immunocompetent and immunocompromised (SUP) individuals grouped in vaccination only (v2–v4 for COMP or v3–v5 for SUP) or by infection and hybrid status (c/c + v). Sera was measured against the ancestral Wuhan isolate, the Delta, Omicron BA.1, or Omicron XBB.1.5 variant. Data are presented as box plots showing the median, mean (+), 25th to 75th percentiles with whiskers, and outliers plotted based on Tukey, the number of samples is indicated above each box plot, 2-way ANOVA followed by Bonferroni's multiple comparisons test for each viral strain separately.

To identify the primary driver of immunity, we compared neutralization titers in individuals with infection, vaccination, or hybrid status. Hybrid immunity, achieved through a combination of vaccination and natural infection, consistently resulted in higher neutralizing titers than either vaccination or infection alone, irrespective of the order of the events (Figure 3*A*). This trend was observed across all viral variants, particularly those with significant antigenic drift from the vaccine's parental strain. The comparison of vaccination strategies in immunocompetent individuals shows a clear advantage of mRNA-based vaccines over vector-based approaches when administered according to guidelines, with an mRNA vaccine boost significantly increasing titers of vector-based vaccinations (ancestral: “a” vs “ab” *P* < .0001; “j” vs “jb” *P* = .0043; 2-way ANOVA with Bonferroni's multiple comparison tests; Supplementary Figure 2*C*). Recent studies demonstrated a superior SARS-CoV-2 vaccine response after heterologous boosting with an unrelated second vaccine [39, 40]. A third mRNA vaccine dose further increases neutralization titers against new variants (ie, Omicron BA.1) (Supplementary Figure 2*C*).

To compare immunocompetent with immunocompromised individuals, we pooled all vaccinated samples (“v”, v2-v4 for COMP, v2-v5 for SUP) and samples from convalescent and mixed immunization status (“c/c + v”). As expected and in agreement with the lower immune competence resulting from the presence of immunosuppressive drugs, all immunocompromised individuals had lower neutralization titers against SARS-CoV-2, regardless of vaccination scheme or infection history. However, our study newly observed that also the immunocompromised participants experienced higher neutralization titers in situations of hybrid immunity (ie, after infection plus vaccination against earlier vaccine variants) (Figure 3*B*). For both Ancestral and Delta virus strains, neutralizing titers in convalescent immunocompromised patients were not significantly lower compared to those in immunocompetent individuals.

Of note, no death occurred during the study period, and overall, only a few patients needed to be hospitalized in the transplant arm of the study cohort (Table 1). Among these, 1 patient needed supplemental oxygen, and 2 required extracorporeal membrane oxygenation/extracorporeal life support treatment. We conclude that, although neutralizing anti–SARS-CoV-2 titers in immunocompromised transplant patients were lower than in immunocompetent individuals, the virus-neutralizing activity was adequate to reduce morbidity and prevent mortality during the observation period.

### Cross-neutralization As a Proxy for Cross-protection

**Figure 4.**
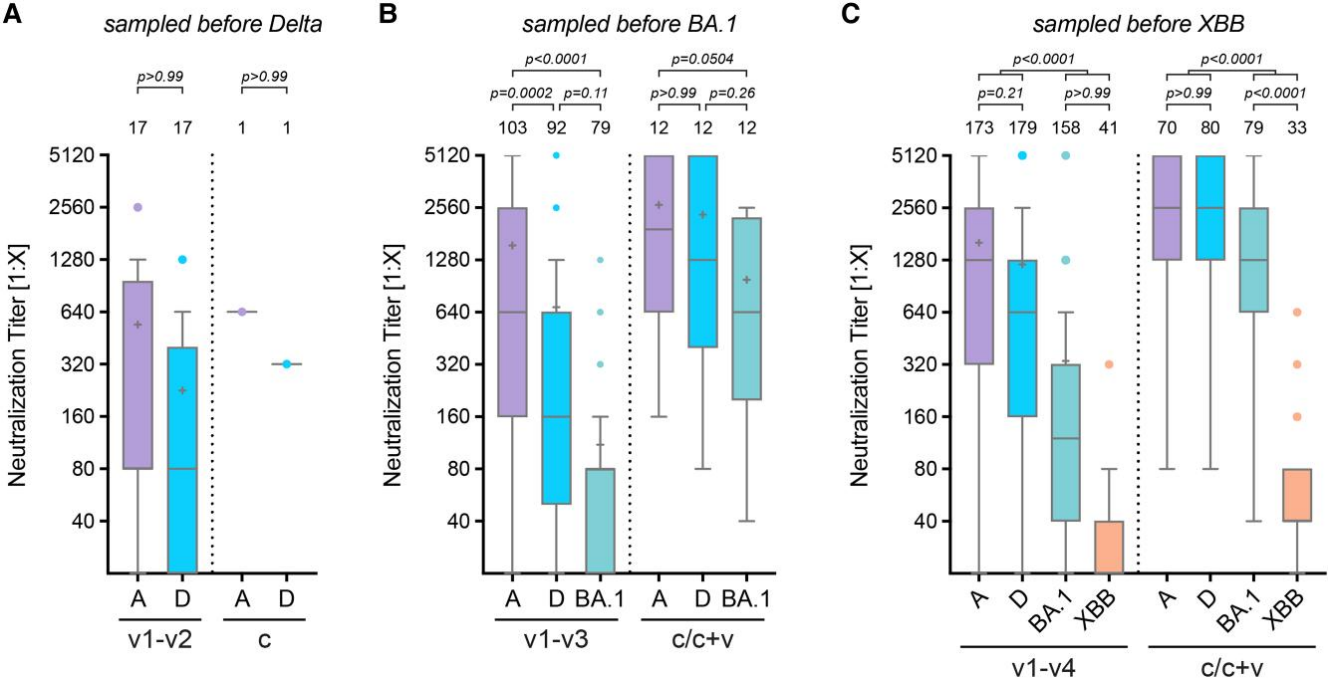
Cross-protection SARS-CoV-2 variants. In vitro cross-protection levels mediated by human sera after 1 to 4 vaccinations (v1–v2, v1–v3, or v1–v4) or by infection and hybrid immunity (c/c + v). Only those sera obtained before the emergence of the respective variant, indicated on top of the graph, were used. Neutralization titers with cross-protection against the Delta (*A*), Omicron BA.1 (*B*), or Omicron XBB.1.5 variant (*C*) after vaccination with or likely exposure to ancestral S or previous variants. Data of immunocompetent samples from 0.5–4 months after vaccination were analyzed. Data are presented as box plots showing the median, mean (+), 25th to 75th percentiles with whiskers, and outliers plotted based on Tukey, the number of samples is indicated above each box plot, 2-way ANOVA followed by Bonferroni's multiple comparisons test.

To assess the cross-neutralization of different SARS-CoV-2 variants, we grouped neutralization data based on the period of the prevalent circulating variant. Accordingly, sera collected before Delta's emergence were tested against ancestral and Delta (Figure 4*A*); those before Omicron BA.1 against ancestral, Delta, and BA.1 (Figure 4*B*), and those before the circulation of Omicron XBB.1.5 against ancestral, Delta, BA.1, and XBB (Figure 4*C*).

When examining a possible cross-protection against different S protein variants, we found that antibodies neutralizing the ancestral virus were principally able to also neutralize Delta (Figure 4*A*), indicating only a low antigenic drift. However, neutralization titers for later variants, such as Omicron BA.1 and XBB.1.5, were significantly lower, demonstrating reduced serum neutralization because of antigenic changes (Figure 4*B* and *C*). The decrease in titer was thereby markedly lower for convalescent individuals when compared to vaccinated, especially for Omicron BA.1, with no significant decrease observed compared to the ancestral or Delta strain (Figure 4*B*).

## DISCUSSION

This study, encompassing >500 specimens from both healthy volunteers and immunocompromised individuals, investigates virus-neutralizing capacity using a live virus neutralization assay. Direct quantification of virus-neutralizing antibodies in human blood is considered the gold standard for assessing antibody-driven immune competence against SARS-CoV-2. We demonstrate that, particularly at higher readout levels, commercial ELISA-based surrogate tests can predict a protective antibody response but do not match the sensitivity of a live neutralization assay.

The study examines the impact of vaccination regimens and virus exposure on the longevity of protective antibodies. Although single vaccine doses were inadequate, 2 shots consistently induced neutralizing responses to the ancestral virus or the Delta variant in immunocompetent individuals. Protection levels against the Omicron variants were moderate or low, reflecting their greater antigenic distance from the vaccine antigen. Remarkably, additional exposure to a circulating virus elevated neutralizing activity, especially against the Omicron variants BA.1 and XBB.1.5, suggesting that viral infection can provide additional protective epitopes, boosting the antiviral response. This effect was recognized early in the pandemic [41].

A notable and somewhat unexpected finding of this study was the similar rise in neutralization/protection levels observed in immunocompromised participants. Early in the pandemic, treatment centers largely based guidance on commercial S-protein binding assays, leading to concerns that protection levels might remain below acceptable thresholds. Although it is clinically mandatory to avoid SARS-CoV-2 infections in this patient group, a relevant outcome of this study was that none of the vaccinated patients who later contracted SARS-CoV-2 required intensive care. Additionally, we observed substantial antibody-based virus neutralization in about 50% of immunocompromised and transplant patients after 4 to 5 vaccine shots, with, as expected, lower absolute levels compared to immunocompetent individuals. Follow-up studies will need to verify whether subsequent SARS-CoV-2 infections can be generally tolerated in this patient group, providing long-term benefits.

All participants in this study received vaccines targeting the ancestral virus. By separating samples into periods with certain prevalent variants, we gained insights into the possible breadth of protection. Samples from the first period (exclusively ancestral virus circulating) neutralized the virus up to the Delta variant, with subsequent infections tending to boost neutralizing capacity. In contrast, sera from the first period hardly neutralized Omicron BA.1 or XBB.1.5 (in the latter case, all titers were <1:160), correlating with the increasing antigenic distance to the vaccine strain. Our study underscored the superiority of hybrid immunity over responses induced by vaccination or SARS-CoV-2 infection alone.

Consistent with earlier suggestions of age dependence in the response to SARS-CoV-2 vaccination, our results from the immunocompetent group confirm the impact of age on protection. Interestingly, among convalescent individuals, no decrease was observed, showing the clear advantage of combined infection and vaccination over mRNA vaccination alone in the elderly population. This held true for all viral variants tested. In the immunocompromised cohort, the absence of an age decrease might be due to the low overall neutralization capacity and/or the limited amount of samples in the subgroups. Furthermore, this study reveals no gender differences and underscores the clear benefits of mRNA vaccines over vector-based vaccines. Additionally, mRNA vaccines exhibit the capacity to enhance low titers induced by vector-based vaccines.

Notably, several samples with no vaccination or infection history had detectable and occasionally high antiviral titers, indicating inapparent or mild COVID-19 infections as confounding for some data. These unnoticed infections are expected to occur with similar likelihood across all groups. This, coupled with a boosting effect, aligns with reports by others [42], including recurrent SARS-CoV-2 infections [43, 44] or infections postvaccination [45].

In summary, our study reveals that none of the vaccinated immunosuppressed study participants experienced severe SARS-CoV-2 infections, and the absence of serious clinical situations strongly supports the suggested practice of reducing immunosuppressive pressure before SARS-CoV-2 vaccination. Given that neutralization titers were significantly lower, new guidelines should consider adjusting the timing and duration of immunosuppressant drug reduction before vaccination. Such a step would likely enhance the production of neutralizing antibodies, which were already detectable at significant levels in most study participants. Furthermore, these results support the need for regular updates to SARS-CoV-2 vaccines to match circulating clinically relevant virus variants for maximal efficacy and suggest that concerns about age-related lower immunity should not be overrated in convalescent individuals.

Although this study includes a larger cohort of immunocompromised individuals than many other studies, the number of samples in various subgroups remains small, making statistical analyses challenging. Nevertheless, we believe the clear findings and trends observed for clinically relevant patient groups will benefit future studies and disease management beyond the SARS-CoV-2 pandemic.

## Supplementary Material

ofae527_Supplementary_Data

## Supplementary Data

Supplementary materials are available at *Open Forum Infectious Diseases* online. Consisting of data provided by the authors to benefit the reader, the posted materials are not copyedited and are the sole responsibility of the authors, so questions or comments should be addressed to the corresponding author.

## Notes

***Acknowledgments*.** The authors are grateful to Alain Amstutz for his helpful comments on the manuscript and thank Nina Khanna for her contribution to enrolling local study participants according to the approved protocol.

***Author contributions***. The study was conceived by D.H., L.U., C.L., C.M., and T.K. with clinical input from W.K. and R.-U.M.; clinical specimens and corresponding data were collected in alignment with the approved study protocol by C.S. and W.K. Experimental procedures were performed by L.U., E.K., Y.Z., C.S., and C.L., and data analysis was conducted by D.H., L.U., C.L., and C.M. The manuscript was jointly written by D.H., L.U., C.M., and T.K., with editing provided by F.O., Y.Z., E.K., M.L., R.-U.M., and W.K. All authors have read and approved the final version of the manuscript.

***Patient consent statement*.** All study participants were enrolled under study-ID No. 2022-00303, approved by the Ethikkommission Nordwest- und Zentralschweiz, and by the Ethikkommission der Ärztekammer Westfalen Lippe (No. 2022-165-b-S) and the Ethikkommission der Ärztekammer Nordrhein (No. 2022060).

***Financial support*.** This research was funded through unrestricted research funds of T.K.
