# Supplementary material for: Benefits of Repeated SARS-CoV-2 Vaccination and Virus-induced Cross-neutralization Potential in Immunocompromised Transplant Patients and Healthy Individuals
Source: Open Forum Infect Dis. 2024 Sep 9;11(10):ofae527. doi: 10.1093/ofid/ofae527 (PMC11450466; doi:10.1093/ofid/ofae527)
Supplement: ofae527_Supplementary_Data [file ofae527_supplementary_data.docx]

Supplementary Materials for:

**Benefits of Repeated SARS-CoV-2 Vaccination and Virus-induced Cross-neutralization Potential in Immunocompromised Transplant Patients and Healthy Individuals**

David Hauser, Lorena Urda, Christopher Lang, Christian Mittelholzer, Fabian Otte, Enja Kipfer, Yuepeng Zhang, Martin Lett, Christiane Schebitz, Roman-Ulrich Müller, Wilfried Klimkait, Thomas Klimkait

**Content:**

Supplementary Method Details

Supplementary Figures

- Supplementary Figure 1: Neutralization Assay Validation
- Supplementary Figure 2: Effect of gender, age, and vaccination type

**Supplementary Method Details**

**Cell lines**

African green monkey kidney cells (Vero E6) were kindly provided by V. Thiel, Bern, Switzerland. Human TMPRSS2 expression in Vero E6 cells (Vero E6-TMPRSS2) was achieved by infecting the cells with a 2nd generation lentiviral vector pLEX307-TMPRSS2-blast (Addgene plasmid #158458) as a transfer vector. After infection, cells were selected in DMEM containing 20µg/mL of Blasticidin for two weeks and analyzed by RT-qPCR for transgene expression. Adenocarcinomic human alveolar basal epithelial cells expressing ACE2 and TMPRSS2 (A549-A/T) were obtained from NIBSC (A549-ACE-2 Clone 8-TMPRSS2; product number 101006). Cells were maintained in high-glucose Dulbecco’s modified Eagle’s medium (DMEM) supplemented with 10% fetal bovine serum (FBS) and 1% penicillin/streptomycin at 37°C with 5% CO_2_. For the neutralization assay, infections were performed in the same media but with 2% FBS. All cell lines were tested negative for mycoplasma.

**SARS-CoV-2 strains and propagation**

Virus stocks were propagated on Vero E6-TMPRSS2 cells cultured in DMEM supplemented with 2% FBS. Of note, we strictly used TMPRSS2-expressing cells for any propagation of SARS-CoV-2 as the expression of TMPRSS2 has been shown to effectively prevent the reported loss of the S1/S2 cleavage site [1]. The infection for virus expansion was done in T175 culture flasks at a cell confluency of about 80%. Medium was aspirated and cells were infected in 4 mL culture medium with 1 mL virus of unknown titer. After 1 hour, 25 mL of culture medium was added to the flask and incubated at 34°C, 5% CO_2_ until most cells had detached. Cell culture supernatant containing virus and floating cells was harvested on day 4 in a 50 mL tube, centrifuged at 1200 x g, 10 minutes, 4°C. 1 mL aliquots were prepared and stored at -80°C for titer determination and neutralization assays. Virus titers were determined by standard plaque-forming assay [2] and reached titer levels between 7*10^5^ and 2*10^6^ TCID50/mL.

All work including infectious SARS-CoV-2 viruses was conducted in a biosafety level 3 facility at the Department of Biomedicine within the University of Basel (approved by the Swiss Federal Office of Public Health [BAG] #A202850/3).

**Live virus neutralization assay (VNA)**

One day before infection A549-A/T or VeroE6 cells were seeded at a density of 8*10^6^ or 4*10^6^ cells per plate (96-well flat bottom), respectively, to reach confluency on the day of infection. Cell culture media (DMEM High Glucose with Stable Glutamine, 2% FBS, 1% Pen / Strep) was used in a final volume of 100 µL / well.

Human sera were serially diluted 1:20 using round bottom 96 well plates, in the first well in a total volume of 200 µL cell culture media. This media/serum mix was further diluted in 1:2 steps by transferring 100 µL to the neighboring well, which already contained 100 µL of cell culture media. The content was thoroughly mixed and 8 such dilution steps were performed. Then, virus inoculum in a volume of 10 µL was added per well and incubated for 1 hour at 34°C, 5% CO_2_. Afterward, 100 µL of this serum/virus mix was added to a cell layer in 100 µL culture media in a second, flat-bottom 96 well plate (a further 1:2 dilution), yielding a 1:40 starting serum dilution on the cells. The accidental addition of 10 µL virus inoculum to the full 200 µL culture volume led to a minimal 5% deviation from the intended 1:40. However, this was considered negligible as this difference had no relevance for the side-by-side comparison of this study.

Culture plates were incubated, depending on plaque development, for 3 to 4 days at 34°C, 5% CO_2_. The respective inoculum amount for the different virus strains used in this study was estimated to yield a comparable number of plaques.

Formaldehyde to a final concentration of 18% was added to each well in a volume of 20 µL / well and incubated for 30 minutes at room temperature. The supernatant was removed and the cells stained with 0.5% Crystal Violet in a volume of 35 µL / well, incubated for 5 minutes, and rinsed with tap water.

Plaques, visible as white spots in the stained plates, were scanned and analyzed with an Immunospot analyzer and the respective automated software (CTL) and, in addition, evaluated macroscopically. The dilution of the first well with at least 50% plaque inhibition compared to the positive control (virus on cells without serum addition on the same plate grown under the same conditions), was reported as the final titer.

**SARS-CoV-2 surrogate virus neutralization assay (sVNA)**

Two surrogate assays were kindly provided by GenScript (SARS-CoV-2 Surrogate Virus Neutralization Test, Cat. #L00847-A) and EuroImmun (NeutraLISA, Surrogate SARS-CoV-2 Neutralization Test, Cat. #El 2606-9601-4) to identify neutralizing antibodies against SARS-CoV-2. The principle of these semi-quantitative tests is that relevant antibodies, present in a given serum sample, would compete with the ACE2 receptor for the binding sites of SARS-CoV-2 S1/RBD proteins, suggesting virus neutralization. Procedures followed the manufacturers’ instructions.

For EuroImmun, samples and controls were diluted 1:5 in ACE2 dilution buffer, incubated for 60 minutes at 37°C in S1 / RBD coated stripes, and washed 3 times. The enzyme conjugate was added for 30 minutes followed by the substrate solution for 15 minutes at room temperature. The reaction was stopped and the absorbance was measured at 450 nm with a microtiter plate reader. % of inhibition was calculated as 100% – (extinction of patient sample × 100% / extinction of blank (mean)), with a positive cut-off above 35% and a borderline reading between 20 and 35%.

For GenScript, samples and controls (pre-diluted 1:10) were diluted 1:2 with the HRP-RBD solution and incubated for 30 minutes at 37°C. The dilutions were added to the hACE2 pre-coated stripes provided for 15 minutes at 37°C. After 4 washing steps, TMB solution was added, turning the color blue. By adding Stop Solution, the reaction was quenched and the color turned yellow. This final solution was read at 450 nm in a microtiter plate reader. The absorbance of the sample is inversely dependent on the titer of the anti-SARS-CoV-2 neutralizing antibodies. % of inhibition was calculated as (1 – (OD value of sample / OD value of negative control) × 100%), with a positive cut-off above 30%.

**ELISA (in-house)**

ELISA plates (high-binding 96-well, Sarstedt, Cat. #82.1581.200) were prepared by blocking with PBS / 3% BSA. The plates were coated with a final concentration of 1.5 µg / mL protein (SARS-CoV-2 Spike [BioLegend, Cat. #793706], Nucleocapsid [University of Dundee, DU 67728], or Membrane [University of Dundee, DU 67699] proteins) and incubated overnight at 4°C. Sera was diluted in PBS / 1% BSA and added to the plate for 2 hours. Plates were washed 3 times with PBS / Tween 0.1%. The secondary antibody (Goat anti-human IgG Peroxidase, Merck, Cat. A0293) was added for 1 hour at a dilution of 1:3000 in PBS / 1% BSA. Plates were washed 3 times with PBS / Tween 0.1%. The ABTS substrate (Invitrogen, Cat. #002024) was added and the absorbance was measured upon development of the colour reaction at 405 nm within 5 to 15 minutes.


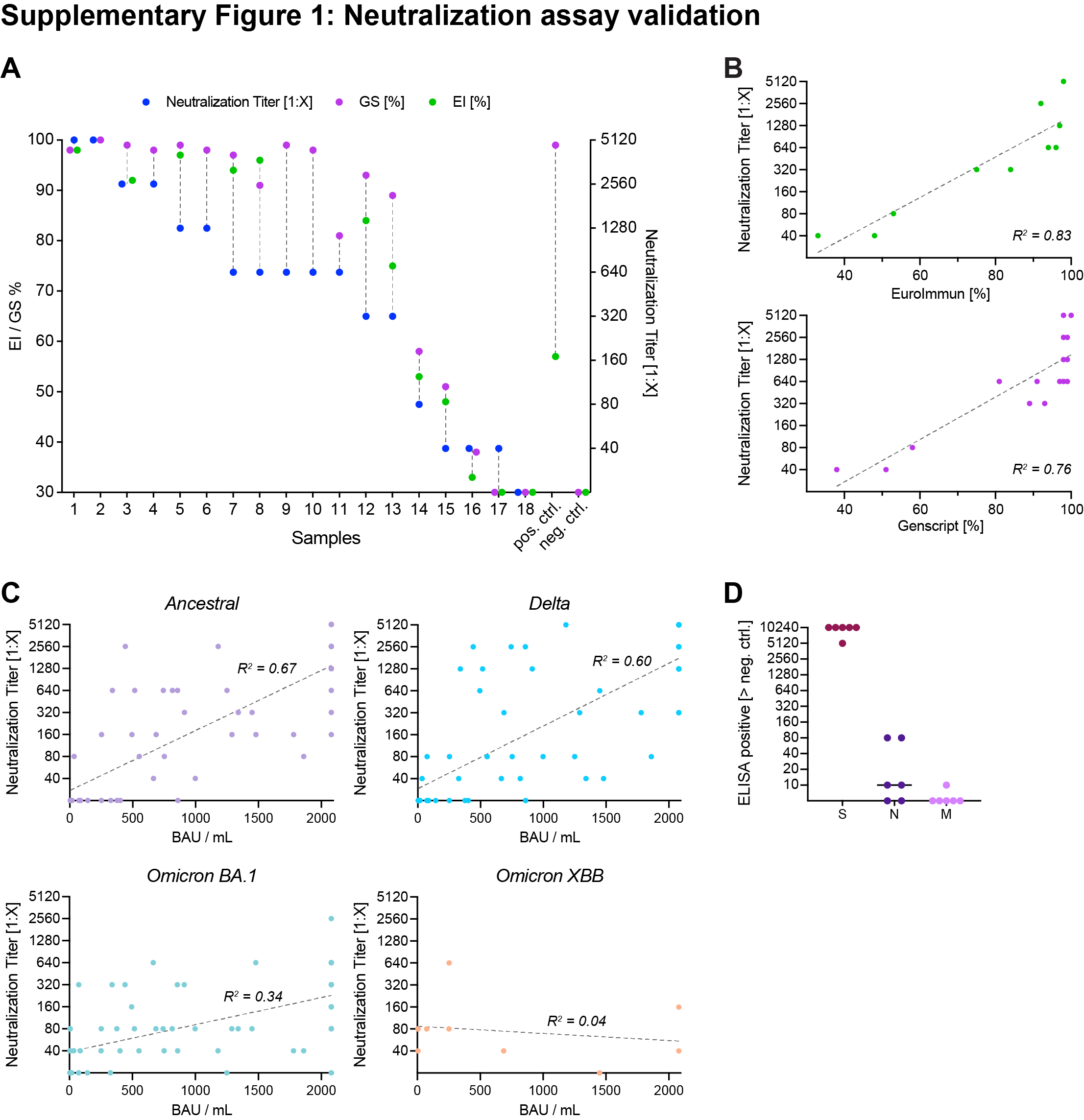


**Supplementary Figure 1: Neutralization assay validation**.

*A*, Comparison of neutralization values of the EuroImmun (EI) and GenScript (GS) tests (in percentage and with a basic cut-off of 30%) to live virus neutralization. *B*, Correlation EI to live virus neutralization (top panel); Correlation GS to live-virus-neutralization (bottom) with *R^2^* indicated. *C*, Correlating DiaSorin ELISA results for the immunocompromised patients to live-virus-neutralization with *R^2^* indicated. For correlation graphs: all timepoints after immunization of a given patient are plotted (Neutralization titer [NT] as a function of BAU / mL). *D*, In-house-ELISA values specific for S, N, or M protein from individuals with 2 or more immunization events. Values depict serum dilutions with an ELISA signal above a threshold defined by negative controls.


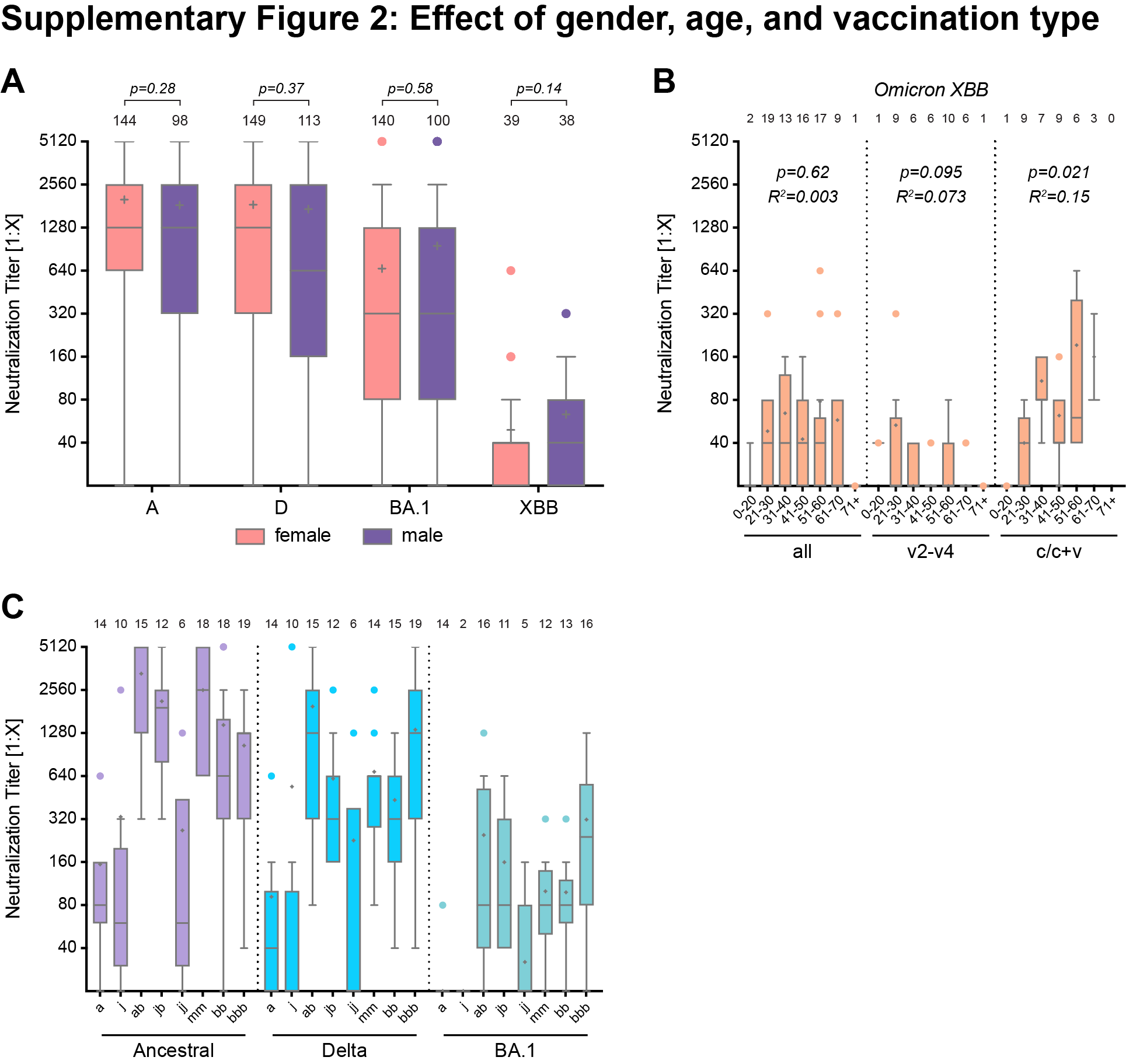


**Supplementary Figure 2: Effect of gender, age, and vaccination type**.

Neutralization titers against SARS-CoV-2 variants 0.5-4 months post-immunization. *A*, Neutralization titer for female and male COMP participants. *B*, Samples grouped in 10-year age intervals for COMP participants for all samples, after 2-4 vaccinations (v2-v4), or after infection or infection and vaccination (c/c+v) measured against Omicron XBB.1.5. *C*, Neutralization titers for individual vaccine combinations (a: AstraZeneca, j: Janssen, b: BioNTech, m: Moderna). Repeated letters indicate repeated vaccinations with the same vaccine. Neutralization titers are plotted for the respective virus strain and age groups as indicated. Data is presented as box plots showing the median, mean (+), 25th to 75th percentiles with whiskers, and outliers plotted based on Tukey; the corresponding number of samples is indicated above each box plot, Mann-Whitney test in (*A*). Linear regression-based *P* values show deviation from slope = 0, the goodness of fit is indicated with *R^2^*.

**References**

1. Sasaki M, Uemura K, Sato A *et al.* SARS-CoV-2 variants with mutations at the S1/S2 cleavage site are generated in vitro during propagation in TMPRSS2-deficient cells. *PLOS Pathogens* 2021;**17**:e1009233.

2. Urda L, Kreuter MH, Drewe J *et al.* The Petasites hybridus CO2 Extract (Ze 339) Blocks SARS-CoV-2 Replication In Vitro. *Viruses* 2022;**14**:106.
